# Comparing Nutritional Values and Bioactivity of Kefir from Different Types of Animal Milk

**DOI:** 10.3390/molecules29112710

**Published:** 2024-06-06

**Authors:** Chiara La Torre, Paolino Caputo, Erika Cione, Alessia Fazio

**Affiliations:** 1 Department of Pharmacy, Health and Nutrition Sciences, University of Calabria, Via Alberto Savinio, 87036 Arcavacata di Rende, Cosenza, Italy; chiara.latorre@unical.it (C.L.T.); erika.cione@unical.it (E.C.); 2 Department of Chemistry, University of Calabria, Via Pietro Bucci, 87036 Arcavacata di Rende, Cosenza, Italy; paolino.caputo@unical.it

**Keywords:** fermentation, microbial population, pH, fatty acid profile, conjugated linoleic acids, phenolics content, antioxidant capacities

## Abstract

The growing interest in fermented dairy products is due to their health-promoting properties. The use of milk kefir grains as a starter culture made it possible to obtain a product with a better nutritional and biological profile depending on the type of milk. Cow, buffalo, camel, donkey, goat, and sheep milk kefirs were prepared, and the changes in sugar, protein, and phenol content, fatty acid composition, including conjugated linoleic acids (CLAs), as well as antioxidant activity, determined by ABTS and FRAP assays, were evaluated and compared. The protein content of cow, buffalo, donkey, and sheep milk increased after 24 h of fermentation. The fatty acid profile showed a better concentration of saturated and unsaturated lipids in all fermented milks, except buffalo milk. The highest content of beneficial fatty acids, such as oleic, linoleic, and C18:2 conjugated linoleic acid, was found in the cow and sheep samples. All samples showed a better antioxidant capacity, goat milk having the highest value, with no correlation to the total phenolic content, which was highest in the buffalo sample (260.40 ± 5.50 μg GAE/mL). These findings suggested that microorganisms living symbiotically in kefir grains utilize nutrients from different types of milk with varying efficiency.

## 1. Introduction

The positive effects of functional dairy products on health have attracted consumer attention and have led to a significant increase in production by the dairy industry, with a focus on probiotics, prebiotics, or symbiotic products. Kefir is one of the foremost probiotic products in the dairy industry. It is a fresh and slightly acidic tasting fermented milk drink produced from kefir grains containing microorganisms in a complex polysaccharide matrix. Cow milk represents the most commonly used milk for industrial kefir production, and it is consumed worldwide. It has long been the primary milk source for human consumption. However, several individuals show hypersensitivity to cow milk proteins. There are several reports that equine, camel, and caprine milks might be preferable to bovine milk with respect to allergenicity, particularly for infants and elderly people. Lajnaf et al. [1] showed that caprine milk proteins are more digestible and more tolerable than bovine milk proteins. Aroua et al. [2] reported that equine milk may reduce digestive and cardiovascular diseases. In the last few decades, there has been increasing consumer interest in drinking raw non-cow unprocessed milks. Cow milk accounts for 82% of the total amount of milk produced in the world, and non-cow dairy species provided 133 million tons in 2016 [3] Buffalo milk is the second most commonly produced milk, comprising 13% of milk globally produced [4], followed by goat (2.3%), sheep (1.4%), and camel (0.2%). Buffalo milk is of great importance due to its nutritional and technological aspects. It has a higher fat, protein, lactose, vitamin, and mineral content than cow milk. It is also whiter and has a thicker consistency, which makes it perfect for the production of fat-based dairy products. Among fresh milks, buffalo milk shows significant levels of total phenolic compounds, as reported by Ahmad et al. [5].

Also, the consumption of donkey milk and its products has been growing. Donkey milk shows similarities to human milk, especially in respect to lipid and protein composition [6]. It contains high levels of polyunsaturated fatty acids and vitamins A, B, and C, low cholesterol, and high levels of lysozyme and lactoferrin, conferring antimicrobial activity [7]. It has high digestibility and a low renal load due to low levels of proteins and inorganic substances, and it can be a good alternative to cow milk in feeding infants or children [3]. Goat milk, on average, contains more fats and proteins, and less lactose than cow milk. Also, goat milk contains less αS1-casein and is less allergenic compared with cow milk [8]. Because of the limited availability of cow milk in developing countries, goat milk and its products are important daily food sources of proteins, phosphates, and calcium [9]. Sheep milk is an excellent source of nutrients in human nutrition; it contains major nutrients and higher levels of total solids compared with goat and cow milks [10], and it is superior to cow milk in the supply of all eight essential amino acids. Camel milk, known as “white gold of the desert”, is mainly consumed raw by the Bedouins, people who inhabit the desert, but both small-scale and large-scale farms for intensive production of camel milk have been implemented worldwide only in recent years [11]. The camel is considered one of the most important dairy animals, and it contributes about 0.3% of the milk produced in the world. Compared with the milk produced by other ruminants, camel milk shows better digestibility in the human gastrointestinal system due to the smallest milk-fat globules and its hypoallergenic properties: camel milk contains more lactoferrin and less allergenic β-lactoglobulin [12]. The allergies to cow milk proteins, together with the desire to taste fermentation products with new flavors, have led some people to use milks of different origins for kefir fermentation. Indeed, in recent years, there has been an increasing trend towards consumption of alternatives to cow kefir. To date, kefir has been successfully made from sheep [13,14], buffalo [15], camel, ewe, donkey, and goat milks [3,16,17,18]. The nutritional and sensory quality, and shelf life of all milks should be improved through fermentation by kefir grains [19]. The grains are composed of an extracellular polysaccharide matrix entrapping a complex mixture of microorganisms belonging to a broad spectrum of species and genera, including lactic acid bacteria (*Lactobacillus*, *Lactococcus*, and *Leuconostoc*), yeast (*Kluyveromyces*, *Candida*, *Saccharomyces*, and *Pichia*), and sometimes acetic acid bacteria (*Acetobacter*) [20], which live in a symbiotic association [21]. The microbial diversity of kefir described in the literature varies greatly. In any case, lactic acid bacteria increase the acidity and viscosity of the fermented beverage by performing a lactic fermentation; the acetic acid bacteria impart fermented milk with a stronger sour taste; yeast ethanol and CO_2_ enrich the flavor of the product. The microorganisms of kefir possess probiotic potential: they exhibit high resistance to the low pH and bile salts in the gastrointestinal tract, and they are able to produce antagonistic substances, such as organic acids and bacteriocins [22], preventing adherence of pathogenic bacteria in the intestinal mucosa [23] and contributing to the improvement of gut health. In recent years, kefir has attracted attention from the scientific community due to numerous health-promoting properties associated with its consumption [1], including physiological, prophylactic, and therapeutic ones, as well as being a safe and inexpensive food that can be easily prepared at home [24]. Therefore, there are several studies reporting the beneficial effects of kefir on animal models and human subjects, including improved digestion and tolerance to lactose [25], antibacterial, hypocholesterolemic, anti-hypertensive, and anti-inflammatory effects [23], control of plasma glucose, and antioxidant, anti-carcinogenic [26], and anti-allergenic activity [27]. Being safe and inexpensive, there is an immense global interest in kefir’s nutritional potential. In fact, the microflora of kefir grains promotes the production of beneficial substances, improving the nutritional profile [23,28]. During fermentation, proteins become easily digestible due to the action of acid coagulation and proteolysis. The lactose from milk is degraded to acid during the fermentation process, which causes pH reduction. Kefir is a good source of Mg, Ca, and P, and, in addition to vitamins in the milk, many vitamins can also be synthesized by LAB during fermentation such as B1, B2, B5, C, and B12 [29]. Finally, several compounds that are generated during fermentation exert a direct influence on the aroma and taste of kefir, such as lactic, acetic, pyruvic, hippuric, propionic, and butyric acids, diacetyl, and acetaldehyde [30]. The lipid content (monoacylglycerols, diacylglycerols, triacyclglycerols, and steroids) in kefir can vary depending on the type of milk. The nutritional composition of kefir as well as its aroma, and rheological, textural, and organoleptic properties widely vary, and are influenced by the quality of raw milk, the origin and composition of the grains, time and temperature of fermentation, and storage conditions [31,32]. Several works have reported the chemical composition of kefir from cow [33], goat, ewe, camel [34], and donkey milks [3], but there is a lack of information in the literature about conjugated linoleic acid (CLA) content of fermented milks obtained from milk of different animal species. CLA is a mixture of geometric and positional conjugated isomers of C18:2, *cis*-9, *cis*-12 linoleic acid. Several in vitro and in vivo studies have highlighted a variety of beneficial health effects, such as anti-carcinogenic, anti-atherosclerotic, antioxidative, anti-obesity, and anti-inflammatory activities, and immune stimulation [35]. CLA naturally occurs in ruminant milk where it is produced as an intermediate component in the ruminal biohydrogenation pathway, carried out by the rumen bacteria. Unfortunately, its nutritional content is comparatively too low to enhance the appropriate physiological effect [36]. A number of food-grade LAB and bifidobacteria were reported to produce CLA in milk products [37,38]. Thus, the increase in CLA content in dairy foods through milk fermentation offers a promising alternative. Although studies have reported the use of food-grade bacteria as a starter [39], and the addition of probiotic culture to develop functional fermented dairy with increased levels of CLA [40], until now, to the best of our knowledge, there are no data in the literature reporting the effect of milk type on the modification of fatty acid profiles and, especially, on the growth of conjugated linoleic acids (CLAs). In this context, the present study aimed at evaluating the influence of milk origin on the chemical composition of the final product in term of protein, sugar content, and fatty acid composition, including CLA, as well as total phenolic content and antioxidant activity. To achieve this objective, cow, buffalo, donkey, camel, goat, and sheep milks were fermented under the same process conditions, using kefir grains with the same biological composition, and the results were compared to determine which type of milk had a better nutritional profile after fermentation.

## 2. Results

### 2.1. Microbial Counts

The microbial diversity of the six types of fermented milks and their kefir grains was analyzed using five different media. The principal microbial groups identified in both milks and grains were total bacteria, *Lactobacilli* (LAB), *Lactococci*, acetic acid bacteria (AAB), and total yeasts (Table 1 and Table 2, respectively).

The results showed that total bacteria were higher in the grains than in the milks, except for the cow sample, which had 2.51 log_10_ cfu/g more total bacteria in the grains than in the corresponding kefir, highlighting that bacterial growth differed according to the type of milk. In all six types of kefir, the main microbial group was AAB, ranging from 2.95 ± 0.05 log_10_ cfu/g in the buffalo sample to 5.77 ± 0.01 log_10_ cfu/g in the camel sample, with the exception of milk from cow and buffalo, in which total bacteria were 1.72- and 1.8-fold higher than the corresponding AAB. Yeasts were absent in camel, buffalo, and sheep fermented milks, but they populated all grains, reaching the highest value in the donkey sample (6.41 ± 0.07 log_10_ cfu/g). *Lactobacilli* preferentially populated fermentation media for camel, buffalo, and goat, and grains for cow, donkey, and sheep. At the end of fermentation, *Lactococci* were predominant in all the kefirs, with the exception of camel and sheep samples, where there was a higher *Lactococci* count in the grains.

### 2.2. pH

**Figure 1:**
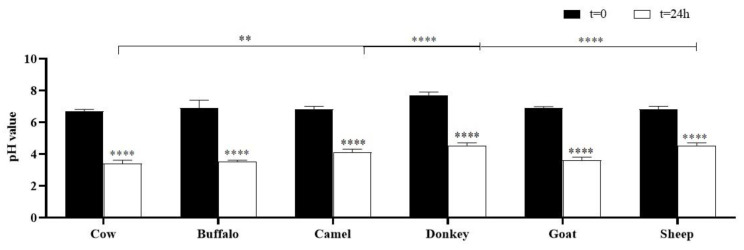
pH values of all samples at time zero and after 24 h of fermentation. Asterisks on the histograms (**** *p* < 0.0001) showed the significance of the pH values of the samples after fermentation compared with the corresponding values at time zero. Asterisks on the lines highlighted the significance of all fermented milks vs. cow kefir (** *p* < 0.01; **** *p* < 0.0001).

The pH values of all samples before and after fermentation are reported in Figure 1. The results showed that there was a significant reduction in pH in all samples (**** *p* < 0.0001) after fermentation compared with the pH values of the corresponding starting milks. The pH values of kefirs ranged from 3.40 ± 0.20 for cow to 4.50 ± 0.20 for donkey and sheep samples. Also, the values obtained after fermentation were compared with cow kefir ones, and significant differences were found for kefirs from camel (4.10 ± 0.20, ** *p* < 0.01), donkey (4.50 ± 0.10, **** *p* < 0.0001), and sheep samples (4.50 ± 0.20, **** *p* < 0.0001).

### 2.3. Protein and Sugar Content

The protein and sugar content of all commercially available milks are listed on the label as part of the nutritional value. These values, after fermentation, were evaluated by Kjeldahl and phenol–sulfuric acid assays, respectively. The results are reported in Table 3, where it is evident that the amount of sugar was reduced by 99.0–99.5% after fermentation, i.e., it was almost zero in all samples. The protein content of cow, buffalo, donkey, and sheep milks increased after 24 h of fermentation by 38.5, 6.2, 29.2, and 28.6%, respectively, while it remained constant in goat and camel samples (3.4 ± 0.1 and 2.8 ± 0.8 g/100 mL). The difference was significant between the cow milk and the corresponding fermented product (*** *p* < 0.001), and between sheep milk and the corresponding kefir (**** *p* < 0.0001). There was also a significant difference in protein content of buffalo and donkey milks compared with reference milk (*** *p*< 0.001 and **** *p* < 0.0001, respectively), and in kefir from camel, donkey, and goat compared with the reference kefir (**** *p* < 0.0001).

### 2.4. Fatty Acid Profile

The qualitative fatty acid profile was determined by comparing the retention times of each peak in the gas chromatography chromatogram with those of the standard compounds. The quantitative composition was evaluated by the external standard method, constructing calibration curves for each identified fatty acid. Fatty acid content, expressed as mg/g, in both unfermented and fermented milks, is reported in Table 4. Among the unfermented milks, the one with the highest amount of identified fatty acids, both saturated and unsaturated, was buffalo milk, followed by camel milk. The main saturated fatty acids in buffalo milk were palmitic (C16:0, 210.1 ± 1.0 mg/g), stearic (C18:0, 172.1 ± 5.3 mg/g), and myristic (C14:0, 155.7 ± 3.3 mg/g), while in camel milk it was myristic acid (C14:0, 111.9 ± 1.0 mg/g). Oleic acid (C18:1n-9) predominated in buffalo milk (231.7 ± 3.3 mg/g), and it was also the most abundant in camel (149.0 ± 0.1 mg/g), sheep (130.7 ± 7.1 mg/g) and goat (77.2 ± 3.3 mg/g) milks. Palmitoleic acid (C16:1n-7) was present in comparable amounts in all the milks studied (52.3 ± 0.1–54.7 ± 0.1 mg/g). Donkey milk demonstrated a lower fatty acid content. The odd fatty acid C17:0 was present in traces in all the samples and it did not change during fermentation, except for buffalo milk, where it significantly increased by 3.2-fold (**** *p* < 0.0001). Also, linoleic acid isomers, 18:2c9t11 and 18:2c10t12, were identified and quantified in the milks. The isomer 18:2c9t11 ranged from 2.8 ± 0.1 mg/g in buffalo milk to 4.4 ± 0.1 mg/g in cow milk, while the isomer 18:2c10t12 was equal to 24.5 ± 0.1 mg/g in camel milk and 36.7 ± 0.8 mg/g in sheep milk, but it was absent in buffalo, donkey, and goat milks. The results showed that the yield of fermented milk was slightly higher than its unfermented counterpart in both unsaturated and saturated fatty acids, except for buffalo milk where SFA, MUFA, and PUFA concentrations remained unchanged. Fermented camel milk showed a significant increase from 1.15 ± 1.1 to 44.1 ± 1.8 mg/g only in palmitic acid (C16:0, **** *p* < 0.0001), whereas fermentation of donkey milk led to a 72-fold increment of stearic acid (C18:0), from 6.3 ± 0.9 to 454.4 ± 3.4 mg/g (**** *p* < 0.0001). The milks that exhibited the greatest increase in fatty acids after fermentation were cow and sheep.

The content of SFA in fermented cow milk was found to be 8.8 times higher than its initial value (**** *p* < 0.0001), while that of MUFA increased 6.5 times (**** *p* < 0.0001). The fermentation of sheep milk resulted in a significant increase in both MUFA and SFA concentrations (**** *p* < 0.0001) by 2.4- and 4.6-fold, respectively. The major fatty acids formed during fermentation in both milks were lauric acid (C12:0), myristic acid (C14:0), palmitic acid (C16:0), and oleic acid (C18:1), whereas stearic acid (C18:0) rose only in sheep milk, from 43.9 ± 0.2 to 191.3 ± 4.1 mg/g. Linoleic acid (C18:2n-6) was 11.7- and 1.5-fold higher in fermented cow and sheep milks, respectively, compared with their corresponding unfermented ones. Linolenic acid (C18:3n-3) significantly increased by 1.4- and 1.8-fold in cow and sheep milks, respectively (**** *p* < 0.0001). Fermented goat milk exhibited an increment of C12:0, C14:0, C16:0, and C18:1n-9 of 4.0, 2.3, 2.1, and 2.0 times, respectively, leading to an increase in SFA and MUFA of 1.9- and 1.6-fold, respectively (**** *p* < 0.0001). During fermentation, the CLA concentration rose at different levels in buffalo (96.5%), goat (90%), and sheep (78.5%) samples (**** *p* < 0.0001), but it decreased in camel milk (** *p* < 0.01). In donkey milk, both CLA isomers were absent before and after fermentation. Of the two CLAs identified, the one that changed during fermentation by increasing its content and affecting the final CLA concentration was C18:2c10t12. Moreover, it was formed in those milks not containing it.

### 2.5. Total Phenolic Content (TPC)

**Figure 2:**
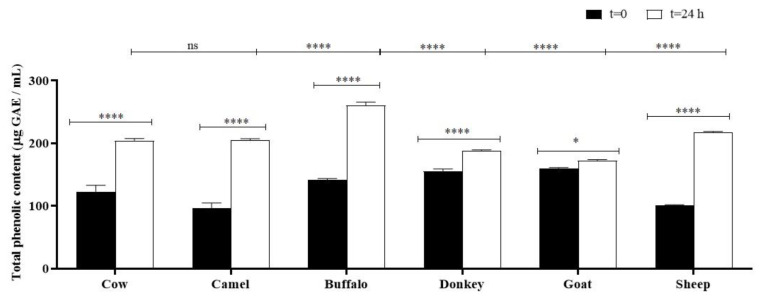
Total phenolic content of unfermented (t = 0, black) and fermented samples (t = 24 h, white). Asterisks on the histograms (* *p* < 0.05 and **** *p* < 0.0001) show the significance of TPC values after fermentation. Asterisks on the lines (**** *p* < 0.0001) highlight the significance of buffalo, donkey, goat, and sheep kefirs vs. cow kefir (control); ns: not significant value.

A Folin–Ciocâlteu assay was carried out on both unfermented and fermented milks (Figure 2). The phenolic content of all samples varied according to the origin of the milks in the range 96.1 ± 8.7 μg GAE/mL, which is the TPC value of camel milk, and 160.2 ± 1.0, which is the TPC value of goat milk. After 24 h of fermentation, TPC values of all samples showed a significant increase (**** *p* < 0.0001) except for goat, which showed increased TPC with a significance of * *p* < 0.05. Donkey milk exhibited a reduction of 44.5% in polyphenol concentration, which was 154. 6 ± 4.0 μg GAE/mL at time zero and 87.6 ± 2.0 μg GAE/mL after fermentation. The best results were found for fermented buffalo (260.4 ± 5.5 μg GAE/mL), camel (204.7 ± 2.5 μg GAE/mL), and sheep milks (218 ± 1.0 μg GAE/mL), which increased by 46, 53, and 54%, respectively, compared with the starting milks (142 ± 2.0 μg GAE/mL, 96.1 ± 8.7 μg GAE/mL, and 101.3 ± 0.5 μg GAE/mL, respectively).

### 2.6. Antioxidants Activity: ABTS and Frap Assays

#### 2.6.1. ABTS Assay

The inhibition percentages of fermented and unfermented milks against the cationic radical ABTS•^+^ are shown in Table 5. Fermented samples highlighted a better antioxidant profile at the highest concentration (333.33 μg/mL_DMSO_), compared with the corresponding unfermented samples, with the exception of the camel and donkey ones for which the inhibition percentage values against the cationic radical did not change significantly (21.7 ± 0.8 and 38.6 ± 4.4; 63.5 ± 4.1 and 62.7 ± 1.1%, respectively, before and after fermentation). Fermented goat and cow milks exhibited the absolute highest %I_ABTS_ at all concentrations, but only at a concentration of 333.33 μg/mL was the significance high (**** *p* < 0.0001) after fermentation in both samples. The inhibition percentage of the goat sample at concentrations of 166.67 and 33.33 μg/mL did not significantly change. Sheep milk exhibited the greatest inhibition percentage increment (57%, **** *p* < 0.0001) only at the highest concentration (333.33 μg/mL).

EC_50_ values, expressed as µg/mL, are reported in Table 6. The reference milk exhibited an EC_50_ value of 365.6 ± 2.6 µg/mL, while goat and donkey milks showed the lowest EC_50_ values (146.7 ± 2.2 µg/mL and 186.7 ± 2.3 µg/mL, respectively) followed by buffalo milk (373.3 ± 2.6 µg/mL). After fermentation, EC_50_ values significantly decreased in all samples (**** *p*< 0.0001), but, among them, fermented cow milk exhibited the lowest EC_50_ value (63.6 ± 1.8 µg/mL), resulting in a reduction of 82.6% compared with the initial value. During the fermentation, the EC_50_ value of sheep milk decreased by 65.5% from the initial value of 1167.0 ± 3.1 µg/mL up to the final value of 402.5 ± 2.6 µg/mL, followed by camel milk (from 1422.0 ± 3.0 µg/mL to 707.1± 2.8 µg/mL) with a EC_50_ reduction of 50%.

#### 2.6.2. FRAP Assay

**Figure 3:**
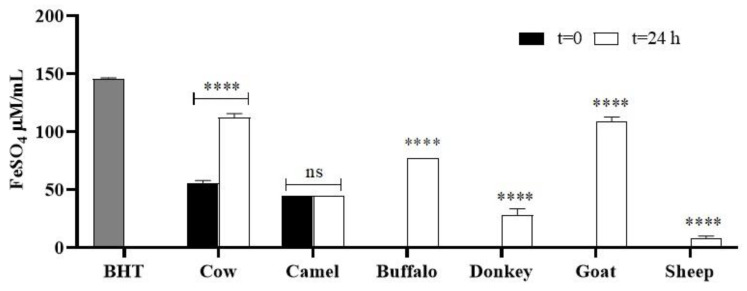
FRAP values (µM/mL FeSO_4_) of fermented and unfermented milks. Asterisks (**** *p* < 0.0001) show the significance of antioxidant activities of samples after fermentation vs. time zero; ns: not significant value.

The ferric reducing antioxidant power (FRAP) assay is a typical ET-based method that measures the reduction of the ferric ion (Fe^3+^)–ligand complex to the intensely blue-colored ferrous (Fe^2+^) complex by antioxidants. Unlike other ET-based methods, the FRAP assay was carried out under acidic pH conditions (pH 3.6) to maintain iron solubility and, more importantly, drive electron transfer. All phenols reduced Fe^3+^ ions to Fe^2+^ ions, which were complexed by 2, 4, 6-tripyridyl-*S*-triazine (TPTZ) at a concentration of 3.3 mg/mL_DMSO_. According to the ABTS assay, the results obtained from the FRAP test again confirmed that fermentation led to an increase in the antioxidant capacity of the samples depending on the milk type (Figure 3). In fact, all the values obtained at time zero improved markedly after fermentation. At time zero, only cow and camel milks had shown positive results with almost comparable values (55.8 ± 2.0 and 44.5 ± 1.0 FeSO_4_ µM/mL, respectively), but, after fermentation, the reducing power of camel milk did not vary while that of the cow sample significantly increased by 50% (**** *p* < 0.0001). Noteworthy were the results related to the reducing power of the goat and buffalo samples, which increased from zero to 108.64 ± 0.64 and 77.1 ± 0.1 µM /mL FeSO_4_ (**** *p* < 0.0001). The FRAP assay confirmed the results of the ABTS assay, showing the best results for goat and cow kefirs. 

### 2.7. Rheology

Fermented and unfermented samples, at the studied concentrations (8 wt%), showed thinning behavior, at the shear rate of 0.1–100 s^−1^. Apparent viscosity decreased with increasing shear rate, meaning that the kefirs behaved as pseudoplastic fluids.

Data reported in Table 7 highlighted that the highest viscosity value among unfermented milks was observed to be 0.312 Pa·s at 0.1 s^−1^, related to the goat milk, while the lowest viscosity value was 0.135 Pa·s for the donkey milk. Among fermented milks, sheep kefir exhibited the highest value of apparent viscosity (0.509 Pa·s at 0.1 s^−1^), whereas buffalo kefir had the lowest value (0.132 Pa·s), resulting in half the initial value (0.246 Pa·s). At a lower shear rate (0.1 s^−1^), the apparent viscosity values of kefir were slightly higher than those of non-fermented milks, resulting in an increase of 52.4, 24.1, 17.2, 18.3, and 63.6% in cow, camel, donkey, goat, and sheep kefirs. Buffalo kefir represented an exception because its initial viscosity decreased by 46.3% after fermentation.

### 2.8. Pearson Correlation

Pearson correlations between antioxidant capacity by ABTS and FRAP assays and proteins, TPC, MUFA, PUFA, and CLA of kefir samples are reported in Table 8. The results highlighted that FRAP values were positively correlated (**** *p* < 0.0001) with TPC, EC_50_ (ABTS), MUFA, and PUFA in cow kefir, and negatively correlated (**** *p* < 0.0001) in the other kefir samples.

## 3. Discussion

Six types of animal milk were fermented by inoculation of activated kefir grains for 24 h at room temperature under aerobic conditions. Fermented milks were investigated for their pH, microbiological population, protein and sugar content, fatty acid profile, TPC, antioxidant activities, and rheological properties. Kefir grains have a complex microbiological composition. *Lactobacilli*, *Lactococci*, yeasts, and acetic acid bacteria have been shown to be present in them. A crude analysis of the grains showed that they are a soft, gelatinous white biological mass of bacteria and yeasts contained in a matrix of proteins and polysaccharides, with a chemical composition of 890 to 900 /kg water, 2 g/kg lipid, 30 g/kg protein, 60 g/kg sugars, and 7 g/kg ash [41,42]. 

The grains represent a microbial symbiotic association since the growth and survival of individual strains are dependent on the presence of each other. Yeasts produce vitamins, amino acids, and other essential growth factors that are essential to maintain the integrity and viability of bacteria, while bacterial metabolic end products are used as energy sources by yeasts [43]. The microbial distribution in the kefir grains influences the population of yeasts and bacteria in the final fermented beverage, and it has been studied, leading to controversial results. Lin et al. assumed that yeasts were located in the inner and intermediate grain zone, with numerous *Lactococci* on the surface area [44]. Therefore, the numbers of yeasts found in the fermented milk were lower than those counted in the grains, while *Lactococci* were predominant in the final drink. On the contrary, other researchers found the yeasts to be distributed in both the outer and the inner grain areas [45]. In the present study, comparative analysis of the microbiota of kefir grains and the corresponding fermented milk showed that both microbial communities were different, as reported by previous studies [46].

The predominant microorganisms identified in both grains and kefirs were total bacteria, *Lactobacilli*, *Lactococci*, acid acetic bacteria, and total yeasts. Although grains with the same microbial composition were used, the results of the distribution of microorganisms between grains and kefir, after 24 h of fermentation, varied according to the type of animal milk. The yeast populations were greater in the grains than in the kefirs, which would suggest that they were not localized on the surface but in the inner layer of grains. The only exception was the cow sample, which exhibited an equal distribution of yeasts in both grains and fermented milk.

Tamine [47] reported that the genus *Lactococcus* tended to grow in milk faster than yeasts. The reason for this tendency was due to its ability to hydrolyze lactose producing metabolites for the survival of yeasts, which in turn synthesized complex B vitamins, hydrolyzed milk proteins, and produced CO_2_ and ethanol [41]. Previous studies highlighted the absence of *Lactococci* on the surface of the grains due to their poor adhesion during growth [48,49].

This study showed that the *Lactococci* population grew differentially in the milks according to their composition, with the exception of camel and goat samples, for which the *Lactococci* count was higher in the grains than in the corresponding milks.

Also, lactic acid bacteria (LAB) were identified in both the grains and in the fermented beverage, where they were differentially distributed according to the type of milk. They were low in the grains cultured in the camel, buffalo, and goat milks compared with their content in the final beverage, while they populated the grains more than the fermented beverages in the case of donkey, cow, and sheep. Similar or even higher LAB populations have been reported in previous studies for kefir grains and their respective drink samples [20].

Previous studies reported that the population of acetic acid bacteria (AAB) in kefir grain was 10^5^–10^7^ [50]. Similar values for AAB in kefir grains were obtained in this study. The results highlighted that the AAB population in the final fermented beverage was lower than in the grains, with the lowest value having been found in buffalo milk. Anyhow, all the fermented milks under study contained an adequate amount of living microorganisms (10^6^ to 10^8^ cfu/g), essential for the products to be considered as probiotics [51].

Among the microbial population, LAB present in the grains produced the β-galactosidase enzyme, causing the degradation of approximately 30% of the lactose to lactic acid as the final product through the Embden–Meyerhof–Parnas (EMP) pathway [52,53].

According to Ismaiel et al. [54], lactose was the predominant sugar in the milk; it was important for the growth of kefir grain microorganisms during the fermentation that involved homolactic and heterolactic LAB in improving the taste of kefir [55]. Homolactic LAB transformed lactose into pyruvic acid, which was converted into lactic acid as the final product of fermentation. Heterolactic LAB led to the formation of additional metabolites such as CO_2_, hydrogen peroxide, acetic acid, and alcohol, along with lactic acid [56], contributing to the aroma and test of the final drink. Lactic acid, the main organic acid produced by LAB, was excreted out of cells and accumulated in the fermentation media, causing a drop in the pH value [57]. Accordingly, the results obtained in this study showed a decrease in pH values depending on the origin of the milk [58]. Accordingly, the lactose content decreased markedly during the first 24 h of fermentation, while β-galactosidase increased [59]. As a result, after 24 h of fermentation, the sugar content was found to be reduced by a factor of 10^−2^ in all kefirs [60].

During the fermentation process, the protein content varied to different degrees according to the type of milk. Proteins of kefir from donkey and buffalo milks slightly increased during fermentation, whereas they significantly increased in cow and sheep samples (**** *p* < 0.001). Goat kefir did not exhibit any change in protein composition. The protein concentration of the final products was generally influenced by the proteolytic activity of the fermenting microorganisms, which quantitatively vary depending on the type of milk. LAB play an important role in milk proteolysis. They are auxotrophic for several amino acids, namely, their growth depends on an exogenous source of amino acids or peptides, which are provided by the proteolysis of casein, the most abundant protein in milks. In general, many LABs contain a cell-envelope proteinase (CEP) that hydrolyzes the proteins into oligopeptides, which are further degraded into shorter peptides and amino acids by intracellular peptidases. They are taken up by the kefir microorganisms or released in the surrounding media [61]. Dallas et al. [62] reported that protein fragments in milk were released in part by kefir microorganisms and in part by native milk proteases. Therefore, the variability of final protein concentrations in fermented milks was not due to a different initial content of casein undergoing hydrolysis, but to a differential release of protein fragments resulting from the activity of both kefir microorganisms and milk native proteases. The findings proved the different proteolytic abilities of starter cultures [63]. Gun [3] reported that kefir from cow and donkey milks contained about 3.45 ± 0.02 and 1.76 ± 0.02% of proteins, respectively. The differences in protein composition between our data and those from the cited sources could be influenced by the minor percentage of inoculated grains, genetic factors, and feeding system [55].

In addition to proteins, LAB also break down the complex milk lipids into free fatty acids due to the lipase activity of LAB, leading to an increase or decrease in them, namely, a different final quantitative and qualitative fatty acid profile for each type of kefir [64,65]. The fermentation of cow, goat, and sheep milks led to a significant increase in SFA, MUFA, and PUFA, while these decreased in camel kefir and remained the same in buffalo kefir. In donkey milk, the fermentation process resulted in an increase in the SFA fraction only (92.2%), leaving MUFA and PUFA levels almost unchanged. In the cow sample, the percentage increases of SFA, MUFA, and PUFA (88.6%, 84.6%, and 54.8%, respectively) were higher than in the goat (49.4%, 37.5%, and 13%, respectively) and sheep (78.2, 57.9, and 33.2%, respectively) samples. The high level of SFA was in accordance with Yadav et al. [65,66], who found SFA yields varying from 72.98 to 90.11 g/100 g in commercial fermented cow milk. Regarding SFA in the cow, goat, and sheep samples, lauric (C12:0), myristic (C14:0), and palmitic (C16:0) acids significantly increased, and only in the sheep and goat samples also stearic acid (C18:0). Lauric acid is known to have antibacterial and antiviral activities [67], while palmitic acid was found to be able to inhibit mutagenesis in a dose-dependent response in bacterial cells in vitro [68]. Among monounsaturated fatty acids, oleic acid exhibited the greatest increments of 94.4% in cow, 50% in goat, and 65.8% in sheep samples. Oleic acid is known to lower plasma cholesterol, LDL (low-density lipoprotein), cholesterol, and triacylglycerols, and to prevent coronary heart disease [69]. Though linoleic and linolenic acids were found in minor amounts compared with oleic acid, their percentage increase during fermentation depended on the type of milk. Cow samples showed the greatest percentage increase in linoleic acid (91.4%), whereas sheep samples had the largest increment of α-linolenic acid (45.6%). 

Cow, goat, and sheep milks showed average PUFA/SFA ratio values of 0.40, 0.20, and 0.27, respectively, while after fermentation these decreased to 0.10, 0.20, and 0.11, respectively. These results were in agreement with those obtained by Stajić et al. [50], who found a reduction in PUFA/SFA values during fermentation. Despite these results, the positive aspect of fermentation resulted from the increment of conjugated fatty acid content, especially in the buffalo sample, where SFA, MUFA, and PUFA decreased, but, in contrast, CLA rose over 95% in the buffalo sample, followed by the goat and sheep samples. 

Viera et al. reported minor concentrations of CLA in cow milk fermented simultaneously by a consortium of the starter culture and the *Lactococcus lactis* subsp. *cremoris* MRS47 strain [70]. Conjugated linoleic acids have been reported to have a wide range of beneficial effects, including anti-carcinogenic, anti-atherogenic, antidiabetic, and immune stimulatory. 

Dairy products are the major natural sources of CLAs, which are produced by specific rumen microorganisms such as *Butyrivibrio fibrisolvens*, as intermediates of the biohydrogenation of linoleic acid to stearic acid, and by desaturation of vaccenic acid (*trans*-11 C_18:1_) in the mammary gland via Δ^9^-desaturase. In addition to rumen bacteria, *Lactobacillus* species can produce CLA from free linoleic acid [71] during milk fermentation by grains. In any case, the increment of CLA depends on the origin of the milks. *Lactobacillus acidophilus* and *Lactobacillus casei* usually use linoleic acid, produced by lipolysis of milk fat, as a substrate for synthesis of CLA, which is then converted to stearic acid (C18:0) [65]. Thus, CLA isomers, which are usually present in relatively low levels in dairy products [52], after their biosynthesis by certain LAB strains enhance the appropriate physiological effect [68].

The phenolic content was evaluated by Folin–Ciocâlteu assay on both unfermented and fermented milks. The results showed that it increased during fermentation, depending on the origin of milk: the highest concentration was found in buffalo kefir, followed by sheep and camel kefirs, and was significantly enhanced compared with the starting value (**** *p* < 0.0001). Data from the literature reported a higher phenolic content in buffalo kefir [72] compared with sheep and camel kefirs [16].

The decrease or increase in phenolic content could be due to the metabolic activity of microorganisms and their capacity to degrade or change the structure of complex phenolic compounds. In fact, some lactic acid bacteria (LAB), such as *Lactobacillus brevis*, *Lactobacillus fermentum*, and *Lactobacillus plantarum*, metabolize phenolic acids by decarboxylation and reduction [14]. The tolerance of lactobacilli to phenolic compounds and their ability to metabolize them are strain or species specific [73,74]. During fermentation, enzymes such as β-glycosidase derived from the fermentative microorganisms are responsible for hydrolyzing complex phenolic compounds to simpler types and the increase in the quantitative amount of TPC [75]. In addition, the phenolic concentration could also decrease during fermentation depending on the origin of the milk [76]. Accordingly, fermentation of donkey milk led to a significant reduction (**** *p* < 0.0001) in phenol by 44% of its initial value, due probably to the degradation action of microbial enzymes in the milk activated during the fermentation. 

Similar results were reported by Yirmibeşoğlu and Öztürk, who found that kefir from donkey milk had less phenolic compound than its non-fermented control [77].

The efficiency of antioxidants in biological systems may differ, and thus two methods based on different principles were used to evaluate the total antioxidant capacity (TAC) of all samples, before and after fermentation: ABTS radical scavenging and ferric reducing antioxidant potential. All kefir samples exhibited varying values in ABTS and FRAP assays depending on the milk type. Fermented milks studied with the ABTS assay showed higher radical scavenging activity than unfermented milks, except for the donkey sample, which showed unchanged values of %I_ABTS_ compared with the starting milk, in accordance with the total phenolic content. Nevertheless, it is noteworthy to point out, as shown by the EC_50_ values, that the antioxidant activity of donkey milk was second only to that of goat milk among the studied milks. It is known that high levels of phenolic compounds in food substrates have always been correlated with high antioxidant activity and free radical scavenging abilities [18]. In the context of kefir fermentation, LAB-based biotransformation had generally led to an increase in the antioxidant activity according to the substrate [19]. In this study, ABTS radical scavenging capacities did not show good correlations with TPC values, with the latter following the order buffalo > sheep > camel ≈ cow > goat > donkey. The results of the ABTS assay for samples were as follows: cow > goat > donkey > buffalo > sheep > camel. Different results were obtained by Baniasadi et al. [16], probably due to differences between microorganisms in kefir grains for the same type of milk. It was reported that the high levels of oligopeptides, peptones, and free amino acids in fermented dairy deriving from microbial proteolytic activity during fermentation led to incremented antioxidant capacity [78]. Although our results showed a significant increase in protein in the cow and sheep samples, the Pearson coefficient showed a positive correlation between them and antioxidant activity (ABTS or FRAP) only for cow kefir. 

Also, the conversion potential of the oxidized form of iron (Fe^3+^) to the reduced form (Fe^2+^) was used to determine the reducing power (RP) of fermented milks. The Fe^2+^ amount can be monitored through spectrophotometry by quantification of Perl Prussian blue at 700 nm. A high absorbance value of the sample indicated a strong RP value. With the exception of the cow sample, for which the RP value doubled after fermentation (**** *p* < 0.001), and the camel sample, which exhibited the same value of RP at times 0 and 24 h, in the other samples of different origins the fermentation process significantly increased the reducing activity compared with the milk values, with a reducing power of 0 (**** *p* < 0.001). Proteins and peptides could be responsible for increased reducing activities after fermentation, as reported by Wang et al. [79,80], but, since there was no simultaneous increase in buffalo and goat proteins during the fermentation, the increased reducing capacity must necessarily be attributed to compounds of a different nature. The ferrous-reducing capacity of cow milk was higher than that reported by Baniasadi et al. [16]. This difference could be attributed to the different microbial populations of kefir grains and the fact that they performed the assay on the methanolic extract of fermented samples and not on the kefir as such.

Fermented and unfermented samples, at the studied concentrations (8 wt%), had shear-thinning behavior, at the shear rate of 0.1–100 s^−1^. Apparent viscosity decreased with increasing shear rate, meaning that the kefir behaved as a pseudoplastic fluid. The Ostwald–de Waele model was successfully used to study the rheological properties of the kefir [32], and it demonstrated that the shear-thinning behavior of kefir samples resulted from the breakdown of the gel structure due to the shear applied to the samples. This type of rheological behavior is common for fermented milk products because of their weak physical bonds, and electrostatic and hydrophobic interactions.

Additionally, this study has shown that the specific type of milk has a considerable effect on the rheological and textural properties of fermented products [81].

Anyway, at a higher shear rate, the viscosity results of kefir were slightly higher than those of non-fermented milks. Buffalo kefir represented an exception because its viscosity decreased by 46.3% compared with that of milk. Different results were reported by Gul et al. [32], who found a higher viscosity and higher consistency index for buffalo kefir than for cow kefir. On the contrary, the high viscosity of sheep kefir compared with that of other samples was justified by its high protein and fat content.

## 4. Materials and Methods

### 4.1. Milks and Reagents

Milk kefir grains were purchased from Kefiralia (Arrasate, Spain). Milks, all of animal origin, were whole cow (Granarolo, Bologna, Italy), whole fresh buffalo (Naples, Italy), and whole goat (Eurospin, Verona, Italy), and three UHT, sheep (EtikèBio, Plouay, France), donkey (Asiplus Bio, Rome, Italy), and camel (NineLife-Europe, Harrow, UK), with all three pasteurized and freeze-dried at 90 °C for 15 min before use. Dimethylsulfoxide (DMSO, ACS grade), methanol (ACS grade), aluminium chloride, acetic acid, hydrochloric acid, sodium acetate, and sodium carbonate were from Carlo Erba Reagent (Milan, Italy). Folin–Ciocâlteu reagent, 2,2-azino-bis (3-ethylbenzothiazoline-6-sulphonic acid) diammonium salt (ABTS), Trolox (6-hydroxy-2,5,7,8-tetramethylchroman-2-carboxylic acid), 2,4,6-Tris(2-pyridyl)-s-triazine (TPTZ) and butylhydroxytoluene (BHT), quercetin, and gallic acid were purchased from Sigma Aldrich (Milan, Italy). The composition of starter cultures obtained by Kefiralia was 10^9^ CFU/g of LAB (*Lactococcus lactis* subsp. *lactis*, *Lactococcus lactis* subsp. *lactis biovar diacetylactis*, *Lactococcus lactis* subsp. *cremoris*, *Leuconostoc mesenteroides* subsp. *cremoris*, and *Lactobacillus kefyr*), *Candida kefyr*, and *Saccharomyces unisporus* subsp., as declared by the producer.

### 4.2. Kefir Grain Activation

Fresh kefir grains were added to cow milk, poured into a glass container, and left to grow at room temperature under aerobic conditions for seven days [82]. Every day, the grains were separated via a plastic sieve and washed three times with deionized water, and then the medium was replaced by fresh milk. 

### 4.3. Fermentation Process

After activation, 10% (*w*/*v*) of grains were separately grown in six types of animal milk for 24 h at room temperature [82]. Then, after the separation of grains, all the fermented milks were freeze-dried (Telstar LyoQuest, Barcelona, Spain) and analyzed to evaluate their protein, sugar, and phenolic content, fatty acid profile, including CLA content, as well as antioxidant activity at zero time and after 24 h of fermentation. The cow sample was used as the reference in all analyses for both milks and kefir because it is the most widely used milk for industrial kefir production.

### 4.4. Microbiological Analysis

Microbiological analyses were performed on both kefir grains and milks after fermentation [83]. Ten grams of the freshly cultured grains were diluted 1:10 with a ringer solution (LAB M Limited, Lancashire, UK), and each mixture was thoroughly homogenized using Stomacher apparatus (Interscience BugMixer^®^ 400 P, Saint Nom, France) for 15 min at maximum speed prior to plating. Ten grams of kefir (devoid of grains) were also diluted in the same way as grains. Then, serial decimal dilutions of all samples from 10^−1^ to 10^−5^ were performed, and 100 μL were inoculated in triplicate, by surface spreading, on specific solid media. The following microorganisms were counted:

i. *Lactobacilli* were plated on MRS (Man Rogosa Sharpe) agar and incubated at 37 °C under anaerobic conditions for 5 days;
ii. *Mesophilic cocci* were plated on M17 agar and incubated aerobically at 37 °C for 5 days;
iii. Acetic acid bacteria (AAB) were plated on GYP medium containing 1% D-glucose, 0.8% yeast extract, 1.5% pepton, and 1.5% agar, and were incubated aerobically at 30 °C for 2 days;
iv. Yeasts and molds were inoculated on PDA medium (potato dextrose agar) and incubated aerobically at 30 °C for 3 days;
v. Total bacteria were plated on PCA medium (plate count agar) and incubated aerobically at 30 °C for 3 days;

Colonies of each type of microorganism were grouped based on color, size, form, elevation, and margin, and the results of the viable counts were expressed as means of logarithm to base 10 of colony-forming units (log_10_ cfu) per gram of sample ± standard deviations.

### 4.5. pH

The pH values before and after fermentation were determined by dipping a previously calibrated pH-meter electrode (Hanna Instruments, Woonsocket, RI, USA) into the samples.

### 4.6. Protein Content Determination According to Kjeldahl Method

Protein content was determined by the Kjeldahl method [84] with the same modification, using a semiautomatic Kjeldahl Apparatus “Velp Scientifica” with two separate parts: 6 digestion blocks and 1 distillation unit. The Kjeldahl method is recognized by Codex Alimentarius as the standard for quantifying milk proteins [7]. The principle of the method is that a strong acid facilitates the digestion of milk so that it releases nitrogen, which can be determined by a suitable titration technique. The procedure involved three-steps, which were digestion, distillation, and titration.

The sample (5 mL) was digested at approximately 430 °C with 98% H_2_SO_4_ (10 mL) in the presence of K_2_SO_4_ (3 g) to raise the boiling point, and CuSO_4_·5H_2_O (300 mg), as a catalyst, to speed up the digestion. The nitrogen in the sample was thus converted to non-volatile ammonium sulfate. After cooling and dilution of the digest, the ammonium sulfate was converted to volatile ammonia gas by heating with 30% NaOH (60 mL). The ammonia was steam-distilled in a receiving flask containing an excess of hydrochloric acid, where it was trapped by forming ammonium chlorate. The residual hydrochloric acid was determined by titration using 0.1 N NaOH. The titration endpoint was determined using an indicator (methyl orange) to estimate the total nitrogen content of the sample. The titration endpoint was detected visually by color change from pink to yellow. The use of a specific conversion factor was needed to convert the measured nitrogen content to the crude protein content (6.37 for milk). 

### 4.7. Sugar Content Determination by Phenol–Sulfuric Acid Assay

The phenol–sulfuric acid colorimetric method was applied to all milk samples in order to evaluate their sugar content [85]. Then, 0.1 mL of samples, previously diluted with distilled water (1:10), was mixed with phenol solution (1 mL, 6% *w*/*v*) and H_2_SO_4_ (1 mL) for 5 min at room temperature, and then left to stir in a boiling water bath for 15 min. A spectrophotometer was used to measure the absorbance of the resulting solution at 490 nm compared with a blank, prepared as described above by replacing 0.1 mL of sample with the same volume of water. The sugar concentration was determined using a calibration curve of glucose, ranging from 0.2 to 1 mg/ mL. The analyses were performed in triplicate.

### 4.8. Fatty Acid Profile

In order to determine the fatty acid profile of milk, samples were derivatized by transesterification into fatty acid methyl esters (FAMEs), according to the method of Lepage et al. [86].

The freeze-dried sample (100 mg) was dissolved in 2 mL of methanol–benzene 4:1 (*v*/*v*) and then slowly added with 200 µL of acetyl chloride, under magnetic stirring, over a period of 1 min, and closed with Teflon caps. The mixture was heated to 100 °C for 1 h, under stirring, and after cooling to room temperature was neutralized with a 6% K_2_CO_3_ solution (5 mL) and centrifuged. Benzene upper phase (100 µL) was diluted with hexane (1:4 *v*/*v*) and the solution was analyzed using gas chromatography (GC). FAMEs were separated using a fused-silica capillary column (Supelcowax™ 10, Milan, Italy) (30 m × 0.32 mm id × 0.25 µm). Helium was used as the carrier gas, with a linear velocity (u) of 37.5 cm/s. The initial temperature was set to 60 °C and held for 5 min, raised to 185 °C at a rate of 16 °C min^−1^ and maintained for 12 min, and subsequently increased to 235 °C at the rate of 20 °C min^−1^. The final temperature was held for 14 min. Temperatures of SPLIT and FID were set to 235 °C.

Peak identification of FAMEs was carried out by comparison with the retention time of the FAME reference standards and confirmed by the fragmentation pattern of gas chromatography–mass spectrometry (GC-MS) chromatograms and library data.

Quantitative determination was performed using a calibration curve for each standard at four different concentrations ranging from 0.1 to 1 mg/ mL. The results, expressed as mg/g, were reported as mean values of three replicates ± standard deviation.

### 4.9. Total Phenolic Content (TPC)

Total phenol content was determined, as described by La Torre et al. [87]. Each experiment was carried out three times, and the results were reported as mean value replicates ± standard deviation (SD) and expressed as μg gallic acid equivalents per mL of sample prepared at a concentration of 100 mg/mL. 

### 4.10. Antioxidant Activity

Antioxidant activity of all samples before (t = 0) and after fermentation (t = 24 h) was estimated using two different methods: ABTS assay and FRAP test. The experiments were performed in triplicate and the results expressed as mean values ± standard deviation (SD). 

#### 4.10.1. ABTS Assay

The ABTS assay was carried out according to a known protocol [88] with some modification. Activated ABTS•^+^ (3 mL), previously diluted with absolute ethanol (1:88), was mixed with 0.1 mL of sample, at different concentrations (10, 5 and 1 mg/mL_DMSO_), and left to incubate for 5 min under magnetic stirring and in the dark. The absorbance was measured at a wavelength of 734 nm and compared with that of Trolox (Trolox equivalent antioxidant capacity, TEAC) solution at the same concentrations of samples. The results were expressed as a percentage of inhibition of radical cation ABTS•^+^ (%I_ABTS_) obtained from a calibration curve of Trolox analyzed in the range of from 0.001 to 1 µg/mL. The EC_50_ values were calculated from the inhibition percentage values using GraphPad Prism 8 software.

#### 4.10.2. FRAP Assay

The FRAP (ferric reducing antioxidant potential) assay was performed using 10 mM TPTZ (15.7 mg TPTZ and 5 mL 40 mM HCl), 250 mM sodium acetate buffer (pH 3.6, 4.10 g of sodium acetate and 16 mL of acetic acid), and 20 mM FeCl_3_ solution [89] in the volumetric ratio 1:10:1. An aliquot (0.1 mL) of each sample (100 mg/mL_DMSO_) was mixed with 2 mL of FRAP reagent and diluted to a volume of 3 mL with H_2_O. The mixture was allowed to stir for 30 min, in the dark, and finally the absorbance of colored complexes was measured at 593 nm. The standard calibration curve was linear at concentrations ranging from 10 to 0.001 g/mL (y = 0.084x − 0.0019, R^2^ = 0.9984).

Results were expressed as µM/mL FeSO_4_ and compared with BHT, used as a positive control.

### 4.11. Rheology

The rheological measurements of fermented milks at a fixed concentration of 8.0 wt% were performed using a strain-controlled rheometer RFS III (Rheometrics, Piscataway, NJ, USA), using a cone and plate geometry with a 50 mm diameter. The cone angle was 0.04 rad and the gap was set at 0.048 mm. The apparent viscosity values, expressed as Pa·s, were recorded in the shear rate range from 0.1 to 100 s^−1^ at 25 °C [90].

### 4.12. Statistical Analysis

All experimental data obtained were expressed as the mean value of three replicates ± the standard deviation (SD). The statistical analyses were performed using GraphPad Prism 9.3.1 software (GraphPad Software, San Diego, CA, USA), and evaluated by two-way ANOVA followed by a Sidak test to make multiple comparisons for pH, sugar, protein, fatty acid, and phenolic content, as well as for EC_50_ (ABTS) and FRAP values, while the Tukey and Dunnett tests were used to evaluate significant differences in %I_ABTS_ and microbial count, respectively. Significance was established at *p* values < 0.05 (*), *p* < 0.01 (**), *p* < 0.001 (***), and *p* < 0.0001 (****). The Pearson correlation coefficient was performed to measure the correlation between the antioxidative capacity values (ABTS and FRAP) and proteins, sugars, SFA, MUFA, PUFA, CLA, and TPC.

## 5. Conclusions

Several works have studied kefir from different animal milks, and the available data in the literature have highlighted their improved nutritional profile compared with the starting milks. A comprehensive screening of kefirs obtained from different milks under the same fermentation conditions was required to find a valid alternative to kefir from cow milk in terms of chemical composition and antioxidant activity.

Therefore, in the present study, we investigated kefir produced from cow, buffalo, camel, donkey, goat, and sheep milks. The findings provided further evidence that the type of milk differentially affected protein content and fatty acid composition, including conjugated linoleic acids, as well as total phenolic content and antioxidant activity of final kefirs. The protein content increased in cow, buffalo, donkey, and sheep samples, while it remained unchanged in goat and camel ones. Fermented milk showed a slight increment in both unsaturated and saturated fatty acids compared with their unfermented counterpart, except for buffalo milk. Cow and sheep samples exhibited the greatest increase in SFA and MUFA concentrations, being the major fatty acids oleic and palmitic. During fermentation, the CLA content in the samples rose at different levels. Of the two CLAs identified, the main isomer was C18:2c10t12, which formed even in those milks not containing it. The absolute highest CLA amount was found in sheep milk, although the maximum percentage increase was found in buffalo milk. The TPC values of all samples increased, with the exception of donkey milk showing a reduction in polyphenol concentration. The best results were found for fermented buffalo milk, followed by camel and sheep milks. All samples highlighted a better antioxidant profile, tested by both ABTS and FRAP assays, compared with the unfermented counterparts, except for donkey milk, which did not undergo any change during fermentation The highest %I_ABTS_ and FRAP values were found for fermented cow milk, followed by goat milk. These results showed that no correlation could be established between the phenol content and the antioxidant activity of the samples, except for cow kefir, as suggested by the Pearson correlation. The antioxidant activities of the kefirs under investigation could be determined by the fact that other biocompounds formed as a result of the action of kefir microorganisms on the nutrients of the source milks. The findings highlighted that sheep fermented milk might be a promising new protein and CLA source as an alternative to cow kefir, which contains the most dominant bovine milk allergen β-Ig, while consumption of fermented goat milk could be associated with good antioxidant activity.

Further research is being performed to evaluate antimicrobial activity, anti-inflammatory properties, and the hypocholesterolemic effect of kefir from different milk sources in order to develop a fermented product with a higher nutritional quality and specific functional properties. Sensory analyses will be carried out on all samples, with the future perspective of commercializing the obtained probiotic drinks.

## Figures and Tables

**Table 1:** Microbial counts expressed as log_10_ cfu/g, carried out on milks after fermentation.

Samples	Total Bacteria	LAB	*Lactococcus*	AAB	Total Yeast
Cow	8.60 ± 0.03 ^a,A^	4.48 ± 0.05 ^b,E^	4.61 ± 0.03 ^b,D^	5.00 ± 0.04 ^b,C^	5.49 ± 0.02 ^a,B^
Camel	4.54 ± 0.01 ^b,B^	2.90 ± 0.04 ^d,D^	4.0 ± 0.08 ^c,C^	5.77 ± 0.01 ^a,A^	0 ^c,E^
Buffalo	5.33 ± 0.01 ^b,A^	3.90 ± 0.01 ^c,C^	4.0 ± 0.05 ^c,B^	2.95 ± 0.05 ^c,D^	0 ^c,E^
Donkey	4.86 ± 0.05 ^b,C^	0 ^e,E^	6.28 ± 0.01 ^a,A^	5.62 ± 0.02 ^a,B^	2.84 ± 0.02 ^b,D^
Goat	4.82 ± 0.06 ^b,B^	4.90 ± 0.13 ^a,B^	6.50 ± 0.02 ^a,A^	3.84 ± 0.02 ^c,C^	2.70 ± 0.02 ^b,D^
Sheep	4.72 ± 0.02 ^b,B^	3.39 ± 0.03 ^c,C^	3.47 ± 0.02 ^c,C^	5.71 ± 0.02 ^a,A^	0 ^c,D^

Values with the same letter were not significantly different (ns), while values with different letters were significantly different. Small letters indicated significance within the same microbial group of kefirs vs. reference kefir (cow); capital letters indicate significance between the different microbial counts in the same sample.

**Table 2:** Microbial counts expressed as log_10_ cfu/g, carried out on grains after fermentation.

Samples	Total Bacteria	LAB	*Lactococcus*	AAB	Total Yeast
Cow	6.09 ± 0.01 ^a,A^	4.78 ± 0.05 ^b,C^	3.30 ± 0.03 ^D^	5.82 ± 0.04 ^b,A,B^	5.76 ± 0.02 ^b,B^
Camel	5.88 ± 0.01 ^b,A^	2.38 ± 0.04 ^d,B^	5.02 ± 0.08 ^b,A^	5.50 ± 0.01 ^b,A^	5.93 ± 0.02 ^b,A^
Buffalo	6.11 ± 0.01 ^a,A^	2.55 ± 0.01 ^d,C^	0 ^d,D^	5.74 ± 0.05 ^b,A^	4.00 ± 0.03 ^d,B^
Donkey	4.74 ± 0.02 ^c,B^	3.74 ± 0.01 ^c,C^	4.28 ± 0.01 ^c,B^	6.35 ± 0.02 ^a,A^	6.41 ± 0.07 ^a,A^
Goat	4.94 ± 0.03 ^c,B^	3.90 ± 0.13 ^c,C^	4.50 ± 0.02 ^c,B^	6.14 ± 0.02 ^a,A^	4.30 ± 0.02 ^c,B^
Sheep	5.63 ± 0.01 ^b,B^	5.41 ± 0.03 ^a,B^	6.14 ± 0.02 ^a,A^	6.70 ± 0.02 ^a^	4.50 ± 0.03 ^c,C^

Values with the same letter were not significantly different (ns), while values with different letters were significantly different. Small letters indicated significance within the same microbial group of kefirs vs. reference kefir (cow); capital letters indicated significance between the different microbial counts in the same sample.

**Table 3:** Protein and sugar content before (t = 0) and after (t = 24 h) fermentation, expressed as g/100 mL ± standard deviation.

Samples	Protein (g/100 mL)		Sugar (g/100 mL)	
	t = 0	t = 24 h	t = 0	t = 24 h
Cow	3.2 ± 0.3 ^b,B^	5.2 ± 0.5 ^a,A^	4.8 ± 0.1 ^a^	0.055 ± 0.007 ^b^
Buffalo	4.5 ± 0.1 ^a,A^	4.8 ± 0.1 ^a,A^	5.1 ± 0.1 ^a^	0.021 ± 0.002 ^b^
Camel	2.8 ± 0.8 ^a,B^	2.4 ± 0.3 ^a,C^	4.3 ± 0.1 ^a^	0.030 ± 0.001 ^b^
Donkey	1.7 ± 0.1 ^a,C^	2.4 ± 0.2 ^a,C^	6.0 ± 0.3 ^a^	0.051 ± 0.003 ^b^
Goat	3.4 ± 0.3 ^a,B^	3.4 ± 0.1 ^a,B^	4.4 ± 0.1 ^a^	0.041 ± 0.005 ^b^
Sheep	3.5 ± 0.4 ^a,B^	4.9 ± 0.1 ^b,A^	2.9 ± 0.1 ^a^	0.023 ± 0.004 ^b^

Values with the same letter were not significantly different, while values with different letters were significantly different. Small letters indicate significance in the same sample, but with different fermentation times, while capital letters indicate significance between the samples and reference milk (cow), and bold capital letters indicate significance between the sample and reference kefir (cow).

**Table 4:** Fatty acid content, expressed as mg/g_,_ of freeze-dried unfermented and fermented milks.

**Unfermented Milk**						
**Fatty Acid**	**Cow**	**Camel**	**Buffalo**	**Donkey**	**Goat**	**Sheep**
C4:0	0.8 ± 0.1 ^A^	0 ^A^	86.4 ± 0.5 ^A^	0 ^A^	0 ^A^	12.1 ± 0.1 ^B^
C12:0	0.4 ± 0.03 ^B^	5.2 ± 0.1 ^A^	38.1 ± 1.2 ^A^	4.05 ± 0.1 ^A^	10.3 ± 0.1 ^B^	9.3 ± 2.2 ^B^
C14:0	13.4 ± 2.2 ^B^	111.9 ± 1.0 ^A^	155.7 ± 3.3 ^A^	4.2 ± 0.7 ^A^	37.2 ± 3.4 ^B^	38.1 ± 4.6 ^B^
C16:0	23.3 ± 3.8 ^B^	1.15 ± 1.1 ^B^	208.3 ± 1.0 ^A^	6.7 ± 2.2 ^A^	66.2 ± 4.8 ^B^	65.3 ± 0.6 ^B^
C17:0	18.9 ± 0.6 ^A^	26.5 ± 0.3 ^A^	18.7 ± 1.2 ^B^	16.7 ± 0.5 ^A^	17.2 ± 0.5 ^A^	16.9 ± 0.1 ^A^
C18:0	17.6 ± 1.9 ^A^	77.6 ± 0.1 ^A^	172.1 ± 5.3 ^A^	6.3 ± 0.9 ^B^	42.1 ± 0.7 ^B^	43.9 ± 0.2 ^B^
C18:1n-9	25.3 ± 0.1 ^B^	149.0 ± 0.1 ^A^	231.7 ± 3.3 ^A^	8.1 ± 3.1 ^A^	77.2 ± 3.3 ^B^	130.7 ± 7.1 ^B^
C16:1n-7	52.5 ± 0.1 ^A^	54.7 ± 0.1 ^A^	52.8 ± 0.2 ^A^	52.6 ± 0.1 ^A^	52.3 ± 0.1 ^A^	52.6 ± 0.1 ^A^
C18:2n-6	2.8 ± 0.3 ^B^	15.3 ± 0.2 ^A^	28.4 ± 1.5 ^A^	2.6 ± 0.5 ^A^	7.2 ± 0.5 ^A^	21.4 ± 0.9 ^B^
C20:4n-6	15.5 ± 0.2 ^A^	17.3 ± 0.1 ^A^	19.1 ± 2.1 ^A^	0 ^A^	15.9 ± 0.1 ^A^	15.8 ± 0.1 ^A^
C18:3n-3	11.7 ± 0.1 ^A^	15.3 ± 0.2 ^A^	11.7 ± 0.1 ^A^	12.1 ± 0.2 ^A^	11.8 ± 0.1 ^A^	12.5 ± 1.1 ^A^
C18:2c9t11	4.4 ± 0.1 ^A^	3.8 ± 0. 1 ^A^	2.8 ± 0.1 ^A^	0 ^A^	4.2 ± 0.1 ^A^	3.5 ± 0.1 ^A^
C18:2c10t12	2.8 ± 0.1 ^A^	24.5 ± 0.1 ^A^	0 ^B^	0 ^A^	0 ^B^	36.7 ± 0.8 ^B^
SFA	74.6 ± 8.8 ^B^	398.5 ± 2.4 ^A^	679.2 ± 12.5 ^A^	37.8 ± 4.3 ^B^	173.2 ± 9.7 ^B^	185.7 ± 8.1 ^B^
MUFA	77.9 ± 4.1 ^B^	203.7 ± 0.1 ^A^	284.6 ± 3.6 ^A^	60.6 ± 3.05 ^A^	129.8 ± 3.3 ^B^	183.3 ± 7.1 ^B^
PUFA	30.1 ± 0.2 ^B^	48.0 ± 0.4 ^A^	59.4 ± 3.7 ^A^	15.0 ± 0.8 ^A^	34.9 ± 0.6 ^A^	49.8 ± 2.1 ^B^
CLA	7.1 ± 0.2 ^A^	28.3 ± 0.2 ^A^	2.8 ± 0.1 ^B^	0 ^A^	4.2 ± 0.1 ^B^	40.3 ± 1.0 ^B^
**Fermented Milk**						
**Fatty Acid**	**Cow**	**Camel**	**Buffalo**	**Donkey**	**Goat**	**Sheep**
C4:0	9.0 ± 0.1 ^A^	0.6 ± 0.2 ^A^	81.1 ± 0.5 ^A^	0 ^A^	0 ^A^	50.2 ± 0.4 ^A^
C12:0	63.8 ± 1.2 ^A^	7. ± 0.5 ^A^	33.1 ± 1.2 ^A^	0 ^A^	41.1 ± 4.2 ^A^	87.1 ± 5.5 ^A^
C14:0	198.5 ± 4.0 ^A^	108.4 ± 6.1 ^B^	141.5 ± 3.3 ^B^	5.2 ± 0.3 ^A^	86.1 ± 7.4 ^A^	202.2 ± 0.7 ^A^
C16:0	350.5 ± 21.8 ^A^	44.1 ± 1.8 ^A^	210.1 ± 1.0 ^A^	8.7 ± 0.3 ^A^	134.5 ± 11.1 ^A^	300.1 ± 7.7 ^A^
C17:0	26.1 ± 0.8 ^A^	17.5 ± 0.1 ^A^	59.5 ± 1.2 ^A^	16.6 ± 0. 1 ^A^	16.5 ± 0.1 ^A^	23.7 ± 0.2 ^A^
C18:0	7.4 ± 1.3 ^A^	23.6 ± 1.0 ^B^	140.3 ± 5.3 ^B^	454.4 ± 3.4 ^A^	64.3 ± 4.5 ^A^	191.3 ± 4.1 ^B^
C18:1n-9	453.7 ± 3.8 ^A^	38.5 ± 2.0 ^B^	218.7 ± 3.3 ^B^	13.1 ± 2.7 ^A^	155.1 ± 12.1 ^A^	382.8 ± 9.1 ^A^
C16:1n-7	52.5 ± 0.1 ^A^	54.6 ± 0.8 ^A^	53.2 ± 0.3 ^A^	52.3 ± 1.4 ^A^	52.7 ± 0.1 ^A^	52.7 ± 0.1 ^A^
C18:2n-6	32.7 ± 3.7 ^A^	15.6 ± 1.6 ^A^	15.5 ± 1.4 ^B^	3.2 ± 0.1 ^A^	12.1 ± 0.9 ^A^	32.8 ± 0.9 ^A^
C20:4n-6	17.3 ± 0.1 ^A^	15.6 ± 0.2 ^A^	17.2 ± 2.1 ^A^	0 ^A^	16.3 ± 0.1 ^A^	18.5 ± 0.1 ^A^
C18:3n-3	16.7 ± 0.4 ^A^	15.6 ± 1.6 ^A^	11.7 ± 0.1 ^A^	12.5 ± 0.1 ^A^	11.5 ± 0.1 ^A^	23.0 ± 0.2 ^A^
C18:2c9t11	3.5 ± 0.1 ^A^	4.1 ± 0.1 ^A^	3.3 ± 0.1 ^A^	0 ^A^	4.1 ± 0.1 ^A^	2.2 ± 0.1 ^A^
C18:2c10t12	5.6 ± 0.1 ^A^	18.4 ± 2.3 ^A^	78.2 ± 0.2 ^A^	0 ^A^	38.0 ± 0.7 ^A^	185.1 ± 5.9 ^A^
SFA	655.2 ± 34.3 ^A^	201.3 ± 12.7 ^B^	665.6 ± 12.5 ^A^	485.2 ± 4.0 ^A^	342.7 ± 27.1 ^A^	854.4 ± 18.7 ^A^
MUFA	506.4 ± 34.8 ^A^	93.1 ± 2.7 ^B^	272.0 ± 3.6 ^A^	65.6 ± 2.8 ^A^	207.6 ± 12.1 ^A^	435.4 ± 9.1 ^A^
PUFA	66.6 ± 4.1 ^A^	47.1 ± 3.5 ^A^	44.4 ± 3.6 ^B^	15.6 ± 0.1 ^A^	40.1 ± 1.0 ^A^	74.5 ± 1.3 ^A^
CLA	9.1 ± 0.2 ^A^	22.5 ± 2.4 ^B^	81.6 ± 0.3 ^A^	0 ^A^	42.1 ± 0.8 ^A^	187.4 ± 6.0 ^A^

Results given as means and standard deviations of triplicate analyses of each sample. SFA = saturated fatty acid, MUFA = monounsaturated fatty acid n-9 = Omega-9, PUFA = polyunsaturated fatty acids, n-6 = Omega-6 fatty acids, n-3 = Omega-3 fatty acids. Values with the same letter were not significantly different, while values with different letters were significantly different. Capital letters indicate significance of each fatty acid between the unfermented and fermented milks.

**Table 5:** %I_ABTS_ of fermented and unfermented milks at three different concentrations (333.33, 166.67, and 33.33 μg/mL).

Samples	Concentrations (μg/mL)					
	333.33		166.67		33.33	
	t = 0	t = 24 h	t = 0	t = 24 h	t = 0	t = 24 h
Trolox	100.0 ± 0.1		50.2 ± 0.1		30.1 ± 0.1	
Cow	50.0 ± 1.4 ^b,A^	74.1 ± 0.6 ^a,A^	25.4 ± 1.4 ^b,B^	59.3 ± 0.5 ^a,A^	17.5 ± 1.4 ^b,A^	51.9 ± 0.5 ^a,A^
Camel	21.7 ± 0.8 ^a,B^	38.6 ± 4.4 ^a,B^	6.6 ± 0.8 ^a,B^	11.4 ± 2.3 ^a,B^	0 ^a,A^	0 ^a,C^
Buffalo	42.8 ± 0.1 ^b,A^	54.8 ± 1.4 ^a,A^	33.3 ± 0.1 ^a,B^	41.0 ± 0.5 ^a,A^	19.3 ± 2.2 ^a,A^	33.1 ± 0.5 ^a,A^
Donkey	63.5 ± 4.1 ^a,A^	62.7 ± 1.1 ^a,A^	41.0 ± 1.4 ^a,B^	46.7 ± 1.4 ^a,A^	26.5 ± 0.5 ^a,A^	30.7 ± 0.5 ^a,A^
Goat	64.0 ± 0.1 ^b,A^	76.2 ± 0.5 ^a,A^	60.1 ± 0.5 ^a,A^	61.2 ± 0.1 ^a,A^	14.7 ± 1.7 ^a,A^	21.2 ± 0.8 ^a,B^
Sheep	30.6 ± 5.1 ^b,A^	71.8 ± 0.8 ^a,A^	0 ^a,B^	0 ^a,B^	0 ^a,A^	0 ^a,C^

Values with the same letter were not significantly different, while values with different letters were significantly different. Small letters indicate significance in the same sample, but with different fermentation times, while capital letters indicate significance between unfermented milks and reference one (cow), and bold capital letters indicate significance between fermented milks and reference one (cow).

**Table 6:** EC_50_ values expressed as µg/mL of fermented and unfermented milks.

Samples	EC_50_ (µg/mL)	
	t = 0 h	t = 24 h
Trolox	1.8 ± 0.2	
Cow	365.6 ± 0.2 ^b,C^	63.6 ± 1.8 ^a,A^
Camel	1422.0 ± 3.0 ^b,E^	707.1 ± 2.3 ^a,F^
Buffalo	373.3 ± 2.6 ^b,C^	211.4 ± 2.3 ^a,D^
Donkey	186.7 ± 2.3 ^b,B^	162.8 ± 2.2 ^a,C^
Goat	146.7 ± 2.2 ^b,A^	109.8 ± 2.1 ^a,B^
Sheep	1167.0 ± 3.1 ^b,D^	402.5 ± 2.6 ^a,E^

Values with the same letter were not significantly different, while values with different letters were significantly different. Small letters indicate significance in the same sample, but with different fermentation times, while capital letters indicate significance between the unfermented samples and reference milk (cow), and bold capital letters indicate significance between the fermented samples and reference kefir (cow).

**Table 7:** Values of apparent viscosity (Pa·s) as a function of shear rate (s^−1^) of unfermented and fermented milks.

Sample	Fermentation Time (h)	Shear Rate (s^−1^)			
		0.1	1.0	10	100
Cow	0	0.171	0.013	0.004	0.003
	24	0.359	0.039	0.008	0.005
Camel	0	0.148	0.007	0.002	0.002
	24	0.195	0.016	0.004	0.003
Buffalo	0	0.246	0.041	0.007	0.003
	24	0.132	0.014	0.005	0.003
Donkey	0	0.135	0.009	0.003	0.002
	24	0.163	0.021	0.006	0.003
Goat	0	0.312	0.028	0.005	0.002
	24	0.382	0.026	0.006	0.003
Sheep	0	0.185	0.008	0.005	0.004
	24	0.509	0.072	0.016	0.008

**Table 8:** Pearson correlations between antioxidant capacity parameters, proteins, phenolic contents, MUFA, PUFA, and CLA of kefir samples.

Cow	Protein	TPC	FRAP	EC_50_	SFA	MUFA	PUFA	CLA
Protein								
TPC	^a^ -							
FRAP	-	^b^ +						
EC_50_	-	-	+					
SFA	-	-	-	-				
MUFA	-	+	+	+	-			
PUFA	-	+	+	+	-	+		
CLA	-	-	-	-	-	-	-	
**Camel**	Protein	TPC	FRAP	EC_50_	SFA	MUFA	PUFA	CLA
Protein								
TPC	-							
FRAP	-	-						
EC_50_	-	-	-					
SFA	-	-	-	-				
MUFA	-	+	-	-	-			
PUFA	-	-	-	-	-	-		
CLA	-	-	-	-	-	-	-	
**Buffalo**	Protein	TPC	FRAP	EC_50_	SFA	MUFA	PUFA	CLA
Protein								
TPC	-							
FRAP	-	-						
EC_50_	-	-	-					
SFA	-	-	-	-				
MUFA	-	-	-	-	-			
PUFA	-	-	-	-	-	-		
CLA	-	-	-	-	-	-	-	
**Donkey**	Protein	TPC	FRAP	EC_50_	SFA	MUFA	PUFA	CLA
Protein								
TPC	-							
FRAP	-	-						
EC_50_	-	-	+					
SFA	-	-	-	-				
MUFA	-	-	-	-	-			
PUFA	-	-	-	-	-	-		
CLA		-	-	-	-	-	-	
**Goat**	Protein	TPC	FRAP	EC_50_	SFA	MUFA	PUFA	CLA
Protein								
TPC	-							
FRAP	-	-						
EC_50_	-	-	-					
SFA	-	-	-	-				
MUFA	-	-	-	-	-			
PUFA	-	-	-	-	-	-		
CLA	-	-	-	-	-	-	-	
**Sheep**	Protein	TPC	FRAP	EC_50_	SFA	MUFA	PUFA	CLA
Protein								
TPC	+							
FRAP	-	-						
EC_50_	-	-	-					
SFA	-	-	-	-				
MUFA	-	-	-	-	-			
PUFA	-	-	-	-	-	-		
CLA	-	-	-	-	-	-	-	

^a^ Negative correlation. ^b^ Positive correlation.

## Data Availability

The data presented in this study are available on request from the corresponding author.
